# Vanadium exposure exacerbates allergic airway inflammation and remodeling through triggering reactive oxidative stress

**DOI:** 10.3389/fimmu.2022.1099509

**Published:** 2023-01-11

**Authors:** Wei Tu, Xiaojun Xiao, Jiahua Lu, Xiaoyu Liu, Eryi Wang, Ruyi Yuan, Rongjun Wan, Yingchun Shen, Damo Xu, Pingchang Yang, Miao Gong, Peisong Gao, Shau-Ku Huang

**Affiliations:** ^1^ Department of Respiratory & Allergy, Third Affiliated Hospital of Shenzhen University, Shenzhen, China; ^2^ The State Key Laboratory of Respiratory Disease for Allergy, Shenzhen Key Laboratory of Allergy & Immunology, Shenzhen University School of Medicine, Shenzhen, China; ^3^ Johns Hopkins Asthma and Allergy Center, Johns Hopkins University School of Medicine, Baltimore, MD, United States; ^4^ Department of Respiratory Medicine, Xiangya Hospital, Central South University, Changsha, China; ^5^ National Institute of Environmental Health Sciences, National Health Research Institutes, Miaoli, Taiwan

**Keywords:** vanadium, house dust mite, airway inflammation, airway remodeling, ROS, vitamin D_3_

## Abstract

**Background:**

Metal components of environmental PM2.5 are associated with the exacerbation of allergic diseases like asthma. In our recent hospital-based population study, exposure to vanadium is shown to pose a significant risk for current asthma, but the causal relationship and its underlying molecular mechanisms remain unclear.

**Objective:**

We sought to determine whether vanadium co-exposure can aggravate house dust mite (HDM)-induced allergic airway inflammation and remodeling, as well as investigate its related mechanisms.

**Methods:**

Asthma mouse model was generated by using either vanadium pentoxide (V_2_O_5_) or HDM alone or in combination, in which the airway inflammation and remodeling was investigated. The effect of V_2_O_5_ co-exposure on HDM-induced epithelial-derived cytokine release and oxidative stress (ROS) generation was also examined by *in vitro* analyses. The role of ROS in V_2_O_5_ co-exposure-induced cytokine release and airway inflammation and remodeling was examined by using inhibitors or antioxidant.

**Results:**

Compared to HDM alone, V_2_O_5_ co-exposure exacerbated HDM-induced airway inflammation with increased infiltration of inflammatory cells and elevated levels of Th1/Th2/Th17 and epithelial-derived (IL-25, TSLP) cytokines in the bronchoalveolar lavage fluids (BALFs). Intriguingly, V_2_O_5_ co-exposure also potentiated HDM-induced airway remodeling. Increased cytokine release was further supported by *in vitro* analysis in human bronchial epithelial cells (HBECs). Mechanistically, ROS, particularly mitochondrial-derived ROS, was significantly enhanced in HBECs after V_2_O_5_ co-exposure as compared to HDM challenge alone. Inhibition of ROS with its inhibitor N-acetyl-L-cysteine (NAC) and mitochondrial-targeted antioxidant MitoTEMPO blocked the increased epithelial release caused by V_2_O_5_ co-exposure. Furthermore, vitamin D_3_ as an antioxidant was found to inhibit V_2_O_5_ co-exposure-induced increased airway epithelial cytokine release and airway remodeling.

**Conclusions:**

Our findings suggest that vanadium co-exposure exacerbates epithelial ROS generation that contribute to increased allergic airway inflammation and remodeling.

## Introduction

The incidence of allergic asthma is increasing worldwide in recent decades (1), and has been considered to be a major global public health concern (2). Environmental pollutants have been suggested to be one of the major risk factors contributing to the increased incidence of asthma (3, 4). Particularly, the metal components of environmental PM_2.5_, such as vanadium, mercury, lead, copper, cadmium and arsenic, have been reported to affect the severity of allergic respiratory diseases (5, 6). Of these, vanadium exposure has been reported to cause occupational bronchial asthma and bronchitis (7, 8). Vanadium levels in air PM_2.5_ have been associated with the increased respiratory symptoms of children (9) and elevated levels of exhaled FeNO (10, 11). We have recently demonstrated that in a hospital-based case-control study, vanadium exposure posed a significant risk for current asthma, particularly for severe asthma, and that its urinary level correlated with the levels of Nε-(hexanoyl)-lysine (HEL; an oxidative stress marker) and circulating IL-8, as well as with the 3- and 7-day accumulated levels of ambient PM2.5 prior to the clinical visit (12). Furthermore, we and others have suggested interactions between environmental pollutants and allergens in the context of respiratory diseases including asthma (13–15). This suggests that vanadium exposure may contribute to the exacerbation of allergic asthma.

As a transition metal, vanadium is widely distributed not only in nature but also in various heavy industries such as steel and petroleum. Incomplete combustion of fuels such as petroleum is the main source of vanadium pollution in nature. It is well-recognized that exposure to an excessive amount of vanadium can cause damage and metabolic changes in multiple systems including respiratory system (16, 17). Recent epidemiological studies have indicated that vanadium exposure may be associated with impaired pulmonary function parameters and reduced asthma control among children with preexisting asthma (18). Studies with mouse models have suggested that exposure to vanadium pentoxide (V_2_O_5_) gives rise to airway smooth muscle thickening, airway mucous cell metaplasia, and an increased proliferation of peribronchiolar myofibroblasts, thereby leading to the development of airway fibrosis and remodeling (19). Mechanistically, exposure to vanadium has been associated with altered DNA methylation of allergic and proinflammatory asthma genes implicated in air pollution related asthma (20). Most interestingly, oxidative stress (ROS) has been suggested as a possible mechanism underlying the vanadium exposure-induced pathological effects (21, 22).

ROS as a central inflammatory mediator has been associated with the aggravation of the occurrence and development of lung diseases (23) and the severity of asthma (24). Of interest, exposure to vanadium has been shown to cause cell apoptosis and mouse lung inflammation through ROS generation (25). Vanadium exposure can also induce oxidative stress and cellular senescence in human lung fibrosis by *In vitro* analysis (26). These findings were supported by similar studies that silica nanoparticles can induce lung inflammation through ROS signaling and its mediated lysosome impairment and autophagy dysfunction (27), and ozone induces lung inflammation through mitochondrial ROS (28, 29). Our recent studies indicated that benzo(a)pyrene (BaP), a ubiquitous environmental pollutant (30), co-exposure significantly induced HDM-induced lung inflammation *via* ROS-mediated release of proinflammatory cytokines (13). These findings raise the possibility that vanadium co-exposure with HDM may exacerbate HDM-induced airway inflammation and remodeling through triggering epithelial ROS generation.

In the present study, we generated asthma mouse model by using either vanadium or HDM alone or in combination and investigated their effects on airway inflammation and remodeling. Especially, we investigated the effect of vanadium co-exposure on HDM-induced epithelial-derived cytokine release and ROS generation by both *in vivo* and *in vitro* analyses. Most importantly, we explored the role of ROS inhibitors and antioxidant vitamin D_3_ (VD_3_) in attenuating the vanadium co-exposure-induced cytokine release and airway inflammation and remodeling.

## Materials and methods

### Mice

Balb/c mice were purchased from Guangdong Medical Laboratory Animal Center (Guangzhou, China). All mice were maintained at Shenzhen University under specific pathogen-free conditions. All experiments were conducted using age- and sex-matched 6- to 8-week-old male and female mice. The experimental protocols were reviewed and approved by the animal Care and Use Committee in Shenzhen University and were in accordance with the guidelines and regulations of the institution.

### Generation of asthma mouse model

**Figure 1:**
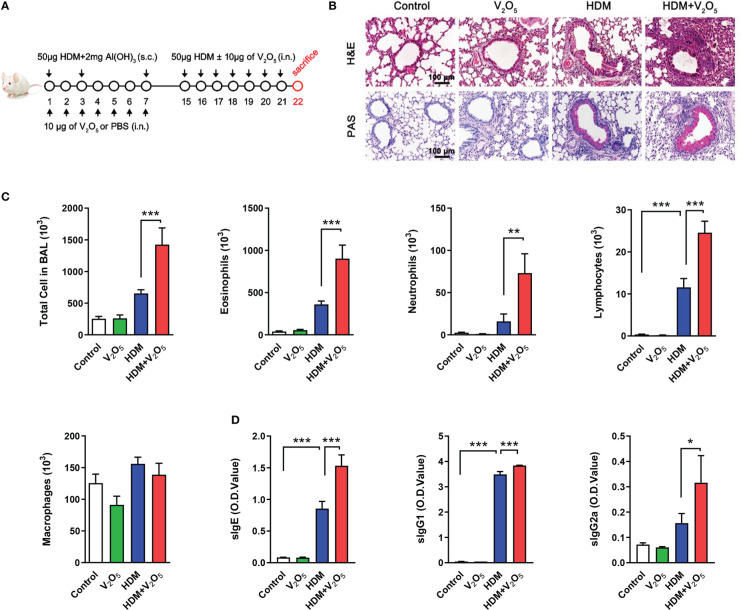
V_2_O_5_ co-exposure exacerbates HDM-induced allergic lung inflammation. **(A)**, Protocol for the generation of mouse model of asthma. Mice were sensitized by intraperitoneal injection with 50 µg of house dust mite extract (HDM) supplemented with 2 mg of Al(OH)_3_ in 200 µl PBS at day 1, 3 and 7. During the sensitization, mice were also administered intranasally every day with or without 10 µg of V_2_O_5_ per mouse in 20 µl of PBS. The mice were challenged intranasally with 50 µg of HDM per mouse in 20 µl of PBS from day 15 to 21 with or without 10 µg of V_2_O_5_ per mouse. All mice were sacrificed 24 h after the last challenge. **(B)**, H&E and periodic acid-Schiff (PAS) staining of lung tissue. **(C)**, Cell differential analysis of Bronchoalveolar lavage (BAL) fluid from mice. **(D)**, ELISAs for levels of HDM-specific IgE, IgG1 and IgG2a in serum. n=7-10/group. Data represent mean ± SEM of two independent experiments. *P < 0.05, ** *P* < 0.01, *** *P* < 0.001.

Mouse asthma model was constructed as previously described and also illustrated in **Figure 1A** (31). Briefly, mice were sensitized by intraperitoneal injection with 50 µg of house dust mite extract (HDM, Greer Laboratories, Lenoir, NC) supplemented with 2 mg of adjuvant aluminum hydroxide (Al(OH)_3_) (Thermo Fisher Scientific, Cleveland, OH, USA) in 200 µl PBS at day 1, 3 and 7. During the sensitization, mice were also administered intranasally every day with or without 10 µg of vanadium pentoxide (V_2_O_5_, the most common form of vanadium; Sigma-Aldrich, Inc., St. Louis, Mo, USA) per mouse in 20 µl of PBS. The mice were challenged intranasally with 50 µg of HDM per mouse in 20 µl of PBS from day 15 to 21 with or without 10 µg of V_2_O_5_ per mouse. All mice were sacrificed 24 h after the last challenge. In some cases, the mice were pre-treated with 100 µg/kg cholecalciferol (vitamin D_3_, Sigma-Aldrich, St. Louis, MO) dissolved in coconut oil (Sigma-Aldrich, St. Louis, MO) *via* oral administration 1 h before every single challenge. PBS-treated age- and gender-matched mice were taken as control.

### Broncheoalveolar lavage fluids

Broncheoalveolar lavage fluids (BALFs) was collected by instillation of 0.8 ml of PBS through a tracheal cannula. BALF samples were centrifuged at 1500 rpm for 5 min at 4°C. Supernatants were collected and stored for cytokine measurements. The number of total cells, eosinophils, neutrophils, lymphocytes and macrophages was determined by flow cytometry as previously described (32). Briefly, cells were blocked with CD16/32 antibody for 15 min, then incubated for 30 min with antibodies as following: anti-Siglet F-PE (S17007L, BioLegend), anti-Mac-3- FITC (M3/84, BioLegend), anti-Gr-1- APC (RB6-8C5, BioLegend), anti-CD3ϵ PerCP-Cyanine5.5 (145-2C11, BioLegend). Cells were analyzed on a FACSCalibur cytometer (BD Biosystems). SSC^high^Siglec-F^+^Mac-3^-^ and SSC^high^Siglec-F^+^Mac-3^+^ cells were identified as eosinophils and alveolar macrophages, respectively. FSC^high^SSC^high^ Gr-1^+^cells were recognized as neutrophils. FSC^low^SSC^low^CD3^+^ cells were identified as lymphocytes.

### Histological analysis

Lung tissues were fixed with 4% of formalin for 24 h. After dehydration, lung tissues were embedded in paraffin, and then cut into 4 µm sections. To evaluate lung inflammation, sections were deparaffinized and dehydrated, and then subjected to H&E and periodic acid–Schiff (PAS) staining. Masson trichrome staining was performed with a kit (Electron Microscopy Sciences, Halfield, PA, USA) under the manufacturer’s instructions.

### ELISA

Concentrations of cytokines (IL-4, IL-5, IL-13, IL-17A, IFN-ƴ, TSLP, IL-25 and IL-33) in cell-free BAL were measured by enzyme-linked immunosorbent assay (ELISA) under the manual instructions of ELISA kits (4A Biotech Co., Ltd., Beijing, China) and (Thermo Fisher Scientific, Cleveland, OH, USA). Serum HDM-specific IgE, IgG1 and IgG2a (sIgE, sIgG1 and sIgG2a) were detected by ELISA as previously described (13). Serum was diluted at 1:4 for IgE, 1:800 for IgG1, and 1:40 for IgG2a.

### Cell culture

Human bronchial epithelial cells (HBECs) were cultured in medium which contains a 1:1 mixture of Dulbecco’s modified Eagle’s medium and Ham’s F-12 (DMEM/F-12; Gibco, Invitrogen corporation, Beijing, China) supplemented with 10% fetal bovine serum (FBS) (Gibco, Invitrogen corporation, Beijing, China) and 1% penicillin-streptomycin (Gibco, Invitrogen corporation, Beijing, China). The cells were maintained at 37°C with a humidified atmosphere containing 5% CO_2_.

### CCK-8 assay

HBE cells were seeded into 96-well plate (5x10^3^/well) and cultured overnight. Then cell viability was measured using Cell Counting Kit-8 (CCK-8; Kumamoto, Japan) according to the protocol. Briefly, cells were treated with different doses of V_2_O_5_ with/without HDM (50 µg/ml) for 24 h. Discard the medium and wash the cells with PBS twice, and then add CCK-8 work solution (diluted with medium, 100 µl/well) to each well of the plate. After incubation at 37°C for 2 h, absorbance at 450 nm was measured with a microplate reader (Thermo Fisher Scientific, Cleveland, OH, USA).

### ROS measurement

ROS in the lung tissues and HBECs were measured by using flow cytometry and immunostaining, respectively. For ROS in lung tissues, lung tissues were minced and then ground slightly. Single-cell suspensions were obtained after filtration with 40 µm of cell strainers (Millipore). Single cells were stained with 5 µM of CM-H_2_DCFDA (Thermo Fisher Scientific) at 37°C for 30 min, and then the ROS generation was measured by flow cytometry. For detection of intracellular superoxide in lung sections, frozen sections were incubated with 5 µM of dihydroethidium (DHE, Thermo Fisher Scientific) 37°C for 30 min, and then fluorescent signal was observed under microscope (Nikon, Chiyoda, Japan). For ROS in HBECs, these treated HBECs were incubated with 5 µM of CM-H_2_DCFDA or 5 µM of mitoSOX (Thermo Fisher Scientific) at 37°C for 20 min. Total and mitochondrial ROS production were analyzed under microscope.

### Immunofluorescence staining

After de-paraffin, dehydration and antigen retrieval, the slides were blocked with blocking buffer at room temperature for 1h. Then tissues were incubated with primary antibodies against α-SMA (Sigma-Aldrich, St. Louis, MO) or vimentin (Cell Signaling Technology, Beverly, MA, USA) at 4°C overnight. After three washes with TBST, secondary antibody incubation was followed at room temperature for 1h. The nuclei were counterstained with 4′,6-Diamidino-2-phenylindole dihydrochloride at room temperature for 10 minutes. The sections were mounted with fluoromount aqueous mounting medium (Sigma-Aldrich, St. Louis, MO) and imaged under microscope (Nikon, Japan). To determine the fluorescent signal, four different high-power fields from each slide were quantified with Fiji (National Institutes of Health) and presented as mean fluorescence intensity per square micrometer.

### Statistical analysis

Statistical significance among experimental groups were analyzed by One-way ANOVA with Student-Newman-Keuls test using GraphPad Prism version 5.1 software (GraphPad Software, San Diego, CA, USA). Data were represented as mean ± SEM. A p-value <0.05 were considered statistically significant for all analyses.

## Results

### V_2_O_5_ co-exposure aggravates HDM-induced airway inflammation

To explore whether vanadium co-exposure can aggravate allergic lung inflammation, we constructed a mouse model of asthma with either HDM or V_2_O_5_ or co-exposure following the protocol as illustrated in **Figure 1A**. Compared to HDM alone, histological analysis demonstrated that mice treated with both HDM and V_2_O_5_ co-exposure showed increased airway inflammation as characterized by dense peri-bronchial inflammatory cell infiltrates (H&E, upper panel) and goblet cell hyperplasia (PAS, lower panel) (**Figure 1B**). The increased airway inflammation caused by V_2_O_5_ co-exposure, but not V_2_O_5_ alone, was further supported by the increased inflammatory cells in BAL samples, including the number of total cells, eosinophils, neutrophils, lymphocytes, and macrophages (**Figure 1C**). Furthermore, serum levels of house dust mite specific IgE (sIgE), sIgG1, and sIgG2a were also increased in V_2_O_5_ co-exposure treated mice as compared to those treated with HDM alone (**Figure 1D**). The results suggest that V_2_O_5_ co-exposure can aggravate HDM-induced airway inflammation.

### V_2_O_5_ co-exposure aggravates HDM-induced epithelial cytokine release

**Figure 2:**
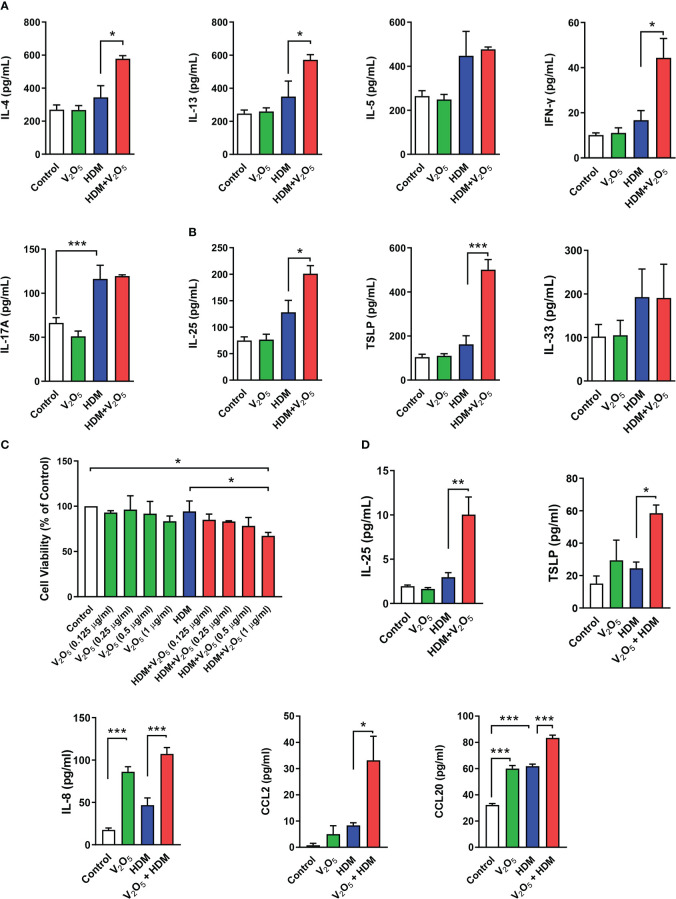
V_2_O_5_ co-exposure aggravates HDM-induced epithelial cytokine release. **(A)**, ELISAs for levels of Th1, Th2, and Th17 cytokines in BAL samples. **(B)**, ELISAs for levels of epithelial-derived cytokines in BAL samples. **(C)**, CCK-8 assay in HBECs treated with HDM (50 μg/ml) or HDM with different doses of V_2_O_5_ (0.125, 0.25, 0.5 and 1 µg/ml) for 24 h. **(D)**, ELISAs for levels of epithelial-derived cytokines and chemokines in supernatants of HBECs treated with either V_2_O_5_ (0.5 µg/ml) or HDM (50 μg/ml) alone or in combination for 24h. A-B: 7-10/group, C-D: three independent experiments. Data represent mean ± SEM. *P < 0.05, ** *P* < 0.01, *** *P* < 0.001.

Next, we investigated whether vanadium co-exposure can aggravate HDM-induced cytokine release. As compared to HDM alone, V_2_O_5_ co-exposure showed significantly increased levels of IL-4, IL-13, and IFNγ in BALFs. No significant increase was noted for IL-5 and IL-17 (**Figure 2A**). Furthermore, we specifically investigated whether V_2_O_5_ co-exposure can aggravate HDM-induced the release of epithelial-derived cytokines. As expected, higher levels of IL-25 and TSLP were observed in BAL samples of V_2_O_5_ co-exposure-treated mice as compared to those treated with HDM alone (**Figure 2B**). While there is no difference in the level of IL-33 (**Figure 2B**). To further confirm whether V_2_O_5_ co-exposure can aggravate HDM-induced epithelial-derived cytokine release, we performed *in vitro* analyses by treating HBECs with either V_2_O_5_ or HDM or in combination. Cell viability was firstly examined by CCK-8 assay in HBECs treated with HDM (50 μg/ml) or HDM with different doses of V_2_O_5_ (0.125, 0.25, 0.5 and 1 µg/ml) for 24 h. As shown in **Figure 2C**, no significant effect on cell viability was noted for HDM-treated HBECs and in HBECs treated with HDM (50 μg/ml) and V_2_O_5_ at a dose from 0.125 to 0.5 µg/ml. A modest reduction in cell viability was observed for V_2_O_5_ co-exposure at a dose of 1 µg/ml. HBECs were then treated with either HDM (50 μg/ml) or V_2_O_5_ (0.5 µg/ml) for 24 h and pro-inflammatory cytokines and chemokines were measured in supernatants by ELISA. Compared to HDM, V_2_O_5_ co-exposure led to increased levels of IL-25, TSLP, IL-8, CCL2 and CCL20 in supernatants of HBECs (**Figure 2D**). Taken together, both *in vivo* and *in vitro* analyses demonstrate that vanadium co-exposure can aggravate cytokine release, particularly those epithelial-derived cytokines and chemokines.

### V_2_O_5_ co-exposure aggravates HDM-induced airway remodeling

**Figure 3:**
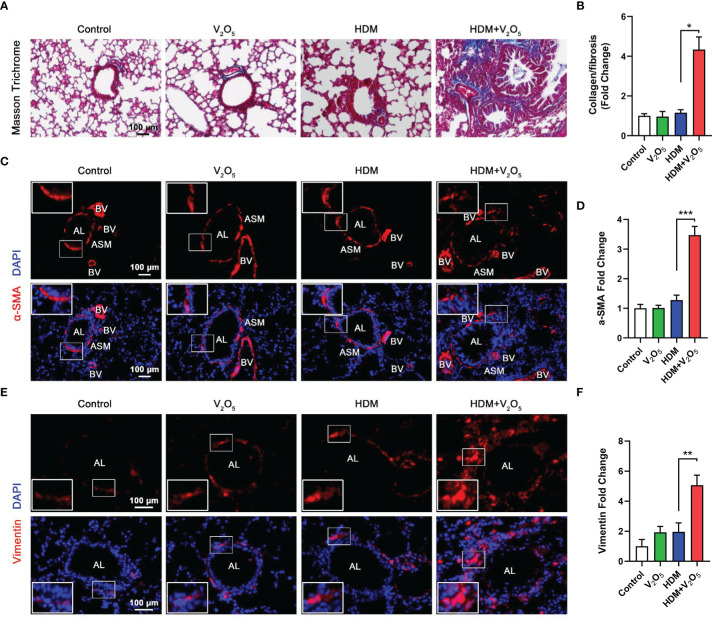
V_2_O_5_ co-exposure aggravates HDM-induced airway remodeling. **(A)**, Masson trichrome staining in the lung tissues. **(B)**, quantification for Masson trichrome staining in **(A)**. **(C)**, Immunofluorescence staining with α-SMA in the lung tissues of mouse model. **(D)**, Quantitative analysis of florescent signals in **(C)**, **(E)**, Immunofluorescence staining with vimentin in the lung tissues of mouse model. **(F)**, Quantitative analysis of florescent signals in **(E)**. AL, airway lumen; ASM, airway smooth muscle; BV, bronchial vein. n=6-8/group. Data represent mean ± SEM of three independent experiments. *P < 0.05, ** *P* < 0.01, *** *P* < 0.001.

Studies have suggested that V_2_O_5_ exposure at 200 µg/animal can lead to the development of airway fibrosis and remodeling in a rat model (19). Thus, we examined whether V_2_O_5_ exposure can aggravate the HDM-induced airway fibrosis and remodeling. Masson trichrome staining was first performed in the lung tissues. Mice with V_2_O_5_ (10µg/mouse) co-exposure showed dramatic increase in airway thickening and collagen deposition/fibrosis as compared to those treated with either HDM or V_2_O_5_ alone (**Figures 3A, B**). The increased airway fibrosis was further confirmed by means of immunofluorescence staining with α-SMA and vimentin. Mice with V_2_O_5_ co-exposure showed a significant increase in the expression of both α-SMA (**Figures 3C, D**) and vimentin (**Figures 3E, F**) in the airways. Taken together, these data suggest that V_2_O_5_ co-exposure can aggravate HDM-induced airway fibrosis/remodeling.

### V_2_O_5_ co-exposure potentiates HDM-induced ROS generation

**Figure 4:**
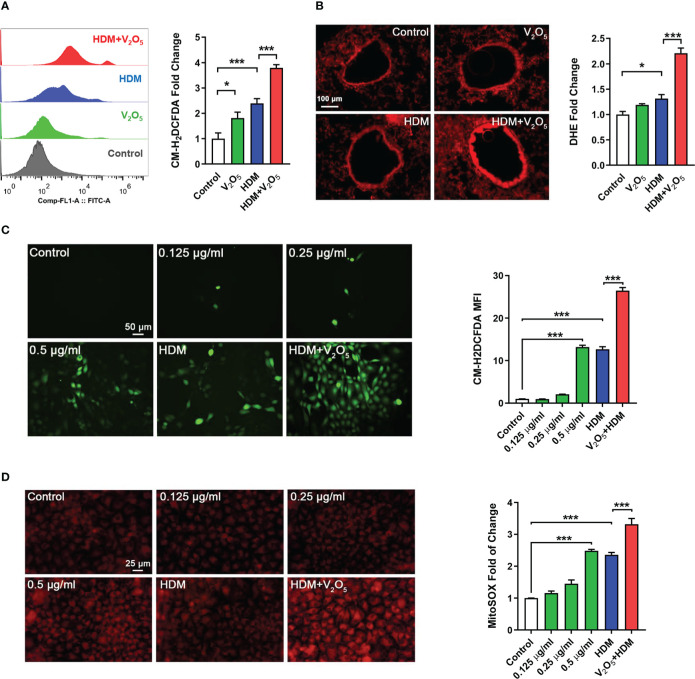
V_2_O_5_ co-exposure potentiates HDM-induced ROS generation. **(A)**, ROS production in single cell from lung tissues of asthma mouse model as detected by flow cytometry analysis with CM-H_2_DCFDA and quantified as fold changes. **(B)**, Representative immunofluorescence images of ROS expression in the lung tissues with dihydrothidium (DHE) immunostaining. **(C, D)**, Representative immunofluorescence images of ROS expression with CM-H_2_DCFDA **(C)** or MitoSOX **(D)** in HBECs exposed to either V_2_O_5_ (0.5 µg/ml) or HDM (50 μg/ml) alone or in combination for 24h. A-B: 5-8/group, C-E: Three independent experiments. Data represent mean ± SEM. *P < 0.05, *** *P* < 0.001.

ROS have been considered as important mediators contributing to oxidative damage and chronic inflammation in allergic diseases (32, 33). To explore the underlying mechanism by which V_2_O_5_ co-exposure aggravates HDM-induced airway inflammation and remodeling, we investigated whether V_2_O_5_ co-exposure could enhance the HDM-induced ROS generation. Firstly, single cells were prepared from lung tissues of asthma mouse model at the end of the treatment/challenge protocol and the relative level of ROS production was evaluated by flow cytometry with CM-H_2_DCFDA. Compared to control, significantly higher level of ROS was found in the HDM group, which was further augmented by V_2_O_5_ co-exposure (**Figure 4A**). The enhanced ROS generation by V_2_O_5_ co-exposure was further supported by the *in vivo* analyses of ROS production in the lung tissues by dihydrothidium (DHE) immunostaining (**Figure 4B**). Lung tissues from V_2_O_5_ co-exposure treated mice had a significant increase in ROS levels as compared to those from HDM-treated mice. These findings were further confirmed by *in vitro* analysis in HBECs. Compared to untreated HBECs, treatment with either HDM or V_2_O_5_ at different doses induced ROS generation as determined by immunostaining with CM-H_2_DCFDA (**Figure 4C**). Notably, the increased ROS were further potentiated by V_2_O_5_ co-exposure with HDM. The same pattern was also observed for mitochondrial ROS, one of the major sources of ROS (33), in those treated and un-treated HBECs (**Figure 4D**). Compared to HDM or V_2_O_5_ treatment, V_2_O_5_ co-exposure induced increased production of mitochondrial ROS as assessed by immunostaining with MitoSOX, a fluorescent mitochondrial ROS reporter dye. Collectively, our findings support the notion that V_2_O_5_ co-exposure potentiates HDM-induced ROS generation, particularly mitochondrial ROS production.

### ROS inhibition alleviates the V_2_O_5_ co-exposure induced cytokine release from epithelial cells

**Figure 5:**
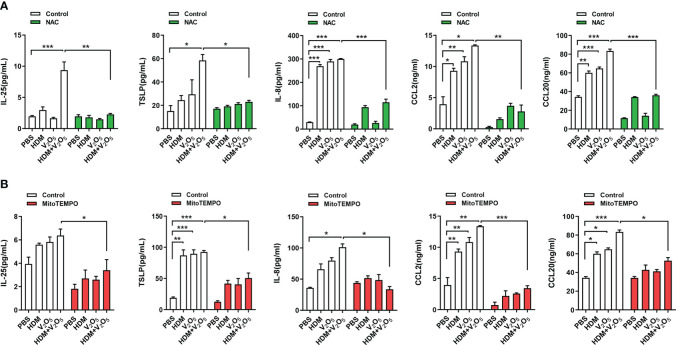
ROS inhibition alleviates the V_2_O_5_ co-exposure induced epithelial cytokine and chemokine release. **(A)**, ELISAs for levels of cytokine and chemokines in supernatants of ROS inhibitor NAC pre-treated HBECs treated with V_2_O_5_ (0.5 µg/ml) or HDM (50 μg/ml) alone or in combination for 24h. **(B)**, ELISAs for levels of cytokine and chemokines in supernatants of mitochondria-targeted antioxidant mitoTEMPO pre-treated HBECs treated with V_2_O_5_ (0.5 µg/ml) or HDM (50 μg/ml) alone or in combination for 24h. Data represent mean ± SEM of three independent experiments. *P < 0.05, ** *P* < 0.01, *** *P* < 0.001.

To examine the role of ROS in V_2_O_5_ co-exposure-induced airway inflammation, we specifically investigated whether ROS play a role in V_2_O_5_ co-exposure induced cytokine release from epithelial cells. HBECs were pre-treated with NAC, a widely used ROS inhibitor, and then treated with either V_2_O_5_ or HDM or co-exposure. Supernatants were harvested after 24 h and cytokines and chemokines in supernatants were measured. Similar to the previous findings that V_2_O_5_ co-exposure significantly upregulated HDM-induced IL-25, TSLP, IL-8, CCL2, and CCL20 production (**Figure 5**). In contrast, pre-treatment with NAC almost completely blocked the V_2_O_5_ co-exposure-induced significant increase in those cytokines and chemokines. The same pattern was also seen when HBECs were pre-treated with mitoTEMPO, a mitochondria-targeted antioxidant (34). These results implicate that vanadium co-exposure induces epithelial cytokine and chemokine release through ROS generation.

### Vitamin D_3_ as an antioxidant alleviates vanadium co-exposure-induced airway inflammation

**Figure 6:**
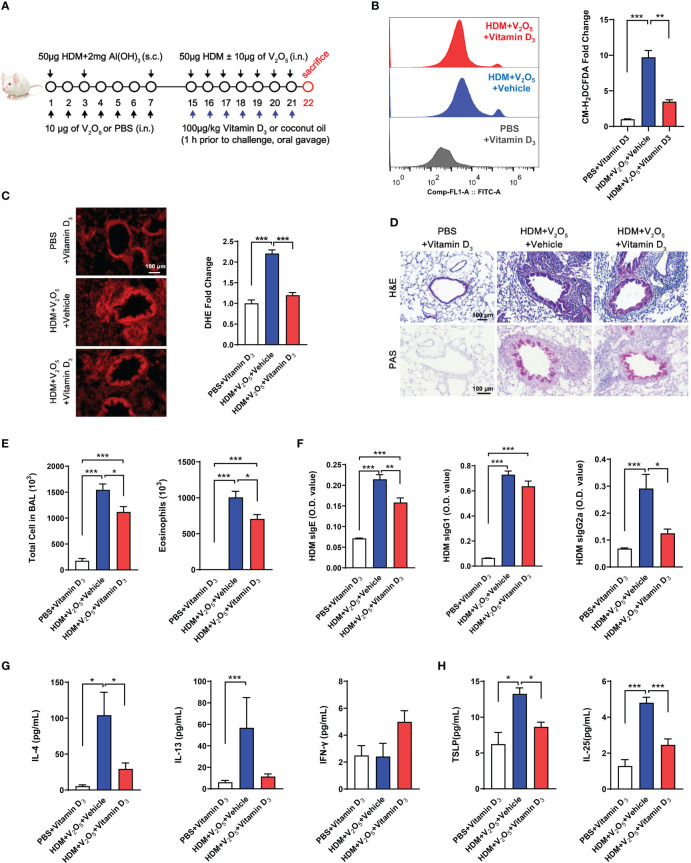
Vitamin D_3_ alleviates vanadium co-exposure-induced airway inflammation. **(A)**, Protocol for the generation of mouse model of asthma. **(B)**, ROS production in single cell from lung tissues of asthma mouse model as detected by flow cytometry analysis with CM-H_2_DCFDA and quantified as fold changes. **(C)**, Representative immunofluorescence images of ROS expression in the lung tissues with dihydrothidium **(DHE)** immunostaining. **(D)**, H&E and PAS staining of lung tissue. **(E)**, Cell differential analysis of Bronchoalveolar lavage (BAL) fluid from mice. **(F)**, ELISAs for levels of HDM-specific IgE, IgG1 and IgG2a in serum. **(G)**, ELISAs for levels of Th1 and Th2 cytokines in BAL samples. **(H)**, ELISAs for levels of epithelial-derived cytokines in BAL samples. n=6-11/group. Data represent mean ± SEM of two independent experiments. *P < 0.05, ** *P* < 0.01, *** *P* < 0.001.

Vitamin D_3_ (VD_3_) supplementation has been shown to protect against oxidative stress processes in the OVA-induced airway inflammation (35). Thus, we hypothesized that VD_3_ could inhibit the V_2_O_5_ co-exposure-induced ROS generation and subsequent airway inflammation. Vitamin D_3_ (VD_3_, 100 μg/kg) was used to treat mice before every single challenge followed the protocol as illustrated in **Figure 6A**. To confirm whether pre-treatment with VD_3_ as an antioxidant can inhibit V_2_O_5_ co-exposure-induced ROS generation, we detected ROS generation in single cell suspension from lung tissues of asthma mouse model by flow cytometry analysis (**Figure 6B**) and in lung tissues by immunofluorescence staining (**Figure 6C**). As expected, both flow cytometry analysis and immunofluorescence staining consistently showed increased ROS generation in V_2_O_5_ co-exposured mice but its level was significantly reduced when those mice were pre-treated with VD_3_. H&E and PAS staining demonstrated that VD_3_ treatment reduced V_2_O_5_ co-exposure induced lung inflammation and mucus production (**Figure 6D**). Significant reductions were noted for the numbers of total inflammatory cells and eosinophils in BAL samples (**Figure 6E**). The same pattern was also noted for serum levels of HDM sIgE and sIgG2a (**Figure 6F**). Similarly, mice pretreated with VD_3_ showed clear reduction for IL-4 in BAL samples as compared to V_2_O_5_ co-exposure-treated mice (**Figure 6G**). In contrast, significant reductions were observed for TSLP, and IL-25 in BAL samples of VD_3_-pre-treated mice compared with V_2_O_5_ co-exposure-treated mice (**Figure 6H**). These preliminary findings suggest that VD_3_ can, at least partially, alleviate vanadium co-exposure-induced cytokine release and airway inflammation.

### Antioxidant vitamin D_3_ inhibits vanadium co-exposure-induced airway remodeling

**Figure 7:**
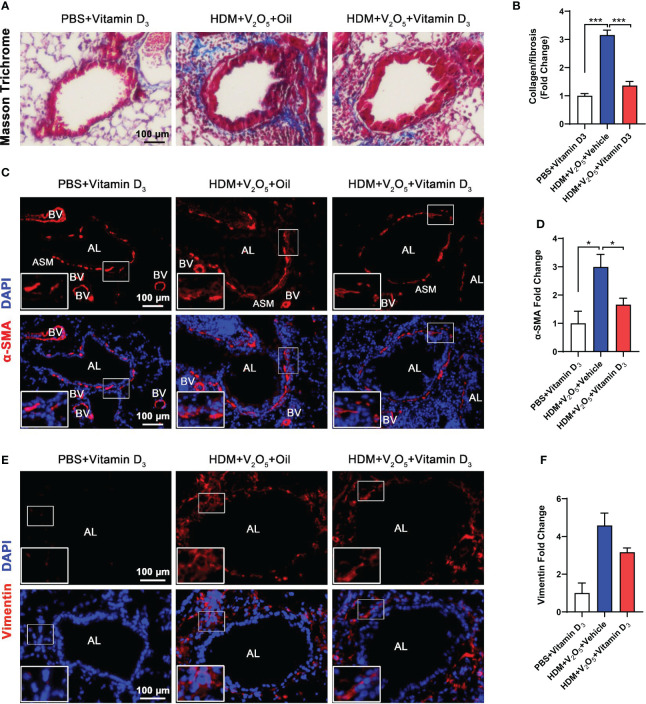
Antioxidant vitamin D_3_ inhibits vanadium co-exposure-induced airway remodeling. **(A)**, Representative immunofluorescence images of airway fibrosis with Masson trichrome staining. **(B)**, Quantification for Masson trichrome staining in **(A)**. **(C)**, Representative immunofluorescence images of α-SMA expression in the lung tissues of mouse model. **(D)**, Quantitative analysis of florescent signals in **(C)**, **(E)**, Representative immunofluorescence images of vimentin expression in the lung tissues of mouse model. **(F)**, Quantitative analysis of florescent signals in **(E)**. n=6-8/group. Data represent mean ± SEM of two independent experiments. *P < 0.05, < 0.01, *** *P* < 0.001.

Next, we investigated whether pre-treatment with VD_3_ could inhibit V_2_O_5_ co-exposure-induced airway fibrosis/remodeling. Masson trichrome staining was performed in the lung tissues. Mice pretreated with VD_3_ showed a significant inhibition of airway thickening and collagen deposition/fibrosis caused by V_2_O_5_ co-exposure (**Figures 7A, B**). The reduced airway fibrosis was further confirmed by immunofluorescence staining with α-SMA and vimentin. Expression of both α-SMA (**Figures 7C, D**) and vimentin (**Figures 7E, F**) was reduced in the airways of VD_3_ pre-treated mice as compared to those treated with V_2_O_5_ co-exposure. Collectively, these data suggest that VD can inhibit vanadium co-exposure-induced airway fibrosis/remodeling.

## Discussion

Evidence is provided here in support of the hypothesis that environmental pollutants coexisted with allergens has the potential to exacerbate airway inflammation and remodeling. In particular, we found that V_2_O_5_ co-exposure enhanced HDM-induced allergic lung inflammation and airway remodeling by in a well-established mouse model of asthma (13, 14). Both *in vivo* and *in vitro* analyses demonstrated that V_2_O_5_ co-exposure amplified HDM-induced epithelial-derived cytokine release. To explore the underlying molecular mechanisms, we found that the V_2_O_5_ co-exposure-aggravated oxidative stress is essential in the V_2_O_5_ co-exposure-induced epithelial cytokine release. Importantly, inhibition of ROS with ROS inhibitors and antioxidant VD_3_ attenuated the V_2_O_5_ co-exposure-induced airway inflammation and remodeling. This study suggests, therefore that ROS play a central role in vanadium co-exposure evoked HDM-induced epithelial cell cytokine and chemokine release contributing to the increased airway inflammation and remodeling.

Recent studies suggest that environmental pollutants co-exposure with allergens may contribute to the increased incidence of asthma (3, 4). Interestingly, DEP alone had no clear effects on the major phenotypes of allergic asthma (36). However, increased allergic responses were found when DEPs were co-exposed with HDM, including airway hyperresponsiveness, upregulated levels of HDM-specific IgE, and recruitment of eosinophils and Th2/Th17 cells (36–38). These findings were supported by our previous studies that benzo(a)pyrene (BaP), a common indoor air pollutant, co-exposure significantly exacerbated Der f 1-induced airway resistance, airway inflammation, and secretion of pro-inflammatory cytokines (13, 14). Vanadium, one of the major components of environmental PM_2.5_, can be found in fresh water, ground water and potable water (<10 nM) (39). The average concentrations of vanadium in the air ranges from 0.1 ng/m^3^ (eastern Pacific) to 0.72 ng/m^3^ (rural northwest Canada) (40). However, industrial production and burning of fuel oil in rural and urban air led to an increased concentration of vanadium in rural and urban air up to 75 ng/m^3^ and 1000 ng/m^3^, respectively (39, 40). Vanadium exposure has been shown to cause occupational bronchial asthma and bronchitis (7, 8), and has been significantly associated with severe respiratory symptoms of children (9) and higher levels of exhaled FeNO (10, 11). Moreover, our recent hospital-based case-control study also revealed that increased vanadium exposure posed a significant risk for current asthma, correlating with the levels of oxidative stress marker, circulating IL-8, and the accumulated levels of ambient PM2.5, providing further environmental and epidemiological evidence (12).

In this study, we found that relatively short-term vanadium exposure alone, similar to DEP (36), had no effect on the major phenotypes of airway inflammation. However, significant exacerbation of allergic airway inflammation was found for vanadium co-exposure with HDM as assessed by inflammatory infiltrates, goblet cell hyperplasia, inflammatory cells in BAL samples, and serum levels of house dust mite specific IgE (sIgE), sIgG1, and sIgG2a. These findings suggest that vanadium co-exposure could aggravate HDM-induced airway inflammation.

Production and release of epithelial-derived cytokines have been considered as one of the early trigger signals to link epithelial injury to downstream inflammatory responses (41). Thus, in addition to Th1, Th2, and Th17 cytokines, we specifically examined epithelial-derived cytokines IL-25, TSLP, and IL-33. As expected, V_2_O_5_ co-exposure induced higher levels of IL-25 and TSLP in BAL samples when compared with those treated with HDM alone. Notably, no difference was observed for IL-33, which may suggest distinct mechanisms for the vanadium co-exposure-promoted epithelial cytokine release. This finding was further supported by *in vitro* analysis. V_2_O_5_ co-exposure significantly enhanced the release of epithelial IL-25, TSLP, IL-8, CCL2 and CCL20 as compared to those treated with HDM alone. These results were also consistent with our previous report that BaP co-exposure can potentiate HDM-induced cytokine release from airway epithelial cells (13). These findings raise the possibility that the increased epithelial cytokine release caused by vanadium co-exposure may contribute to the enhanced Th2-associated airway inflammation in asthma.

Further, we explored the possible mechanisms regarding the vanadium co-exposure-induced cytokine release. ROS have been considered as central inflammatory mediators that contribute to oxidative damage, chronic inflammation in allergic diseases (32, 33, 42–44), and the severity of asthma (24). Our previous studies have also suggested that BaP co-exposure significantly induced HDM-induced lung inflammation *via* ROS-mediated release of proinflammatory cytokines (13). Thus, it is possible that vanadium co-exposure with HDM may exacerbate HDM-induced ROS generation, leading to epithelial cytokine release and subsequent airway inflammation. Indeed, exposure to vanadium has been shown to induce ROS generation that contribute to cell apoptosis, barrier dysfunction and subsequent airway inflammation (25, 45–47). Furthermore, *in vitro* analysis with also provide evidence that vanadium treatment can evoke oxidative stress and cellular senescence in human lung fibroblasts (26). In this study, we provided evidence that vanadium co-exposure potentiated HDM-induced ROS generation, particularly mitochondrial ROS production by both *in vivo* and *in vitro* analyses. To further determine the role of ROS in vanadium co-exposure-induced airway inflammation, we specifically investigated its role in vanadium co-exposure induced epithelial cytokine and chemokine release. Notably, pre-treatment with both NAC as a ROS inhibitor and mitoTEMPO as a mitochondria-targeted antioxidant (34) almost completely blocked the vanadium co-exposure-induced cytokine and chemokine release from human primary airway epithelial cells. These findings further support the notion that ROS play a critical role in oxidative damage and epithelial cytokine and chemokine release. Mechanistically, we have provided evidence that ROS regulate allergic airway inflammation and asthma through controlling either autophagy/mitophagy (48) or NLRP3 inflammasome activation (49). Thus, it is likely that the ROS-mediated autophagy/mitophagy or NLRP3 inflammasome plays an important role in vanadium co-exposure-induced allergic airway inflammation and asthma, which warrant a further investigation in the future.

Vitamin D_3_ has been reported to have a broad range of biological activities, including nutrition, anti-infective effect, and antioxidant stress function (35, 50, 51). VD_3_ supplementation is a relatively cheap, easy and non-invasive intervention in asthma management. VD_3_ supplementation has been associated with reduced incidence of asthma attacks (52) and severe exacerbation (53). Increased serum levels of VD_3_ have been shown to correlate with better pulmonary function and improved quality of life in pediatric asthmatics (54). Recent study has proved experimental evidence that VD_3_ supplementation protects against oxidative stress in the OVA-induced mouse model of asthma (35). In this study, we provided new evidence that pretreatment with vitamin D_3_ could, at least partially, inhibit V_2_O_5_ co-exposure-induced ROS production and airway inflammation. Particularly, both TSLP and IL-25 showed statistically significant reduction in vitamin D_3_ treated mice with V_2_O_5_ co-exposure. These findings implicate that VD_3_ supplementation could be used routinely to prevent lung tissue oxidative damage and inflammation caused by environmental pollutants co-exposure with allergens.

In addition to airway inflammation, we have also examined the effects of vanadium co-exposure on HDM-induced airway fibrosis/remodeling in these mouse model of asthma. Recent studies have changed our understanding of asthma as purely an inflammation and suggested that alterations within the structural cells of the airway (airway remodeling), together with abnormal immune responses, lead to the full manifestation of asthma (55–58). Airway remodeling is a gradual progression in symptoms and irreversible decline in lung function due to the structural changes in the airway walls (59–61). Studies have demonstrated that vanadium exposure can lead to the development of airway fibrosis and remodeling (19). Our study provided further supporting evidence that vanadium co-exposure could significantly potentiate HDM-induced airway remodeling, and importantly, pre-treatment with VD_3_ could reverse such effects. This offers a great promise to prevent/inhibit environmental pollutant-evoked allergen-induced fibrotic remodeling and development of asthma.

**Figure 8:**
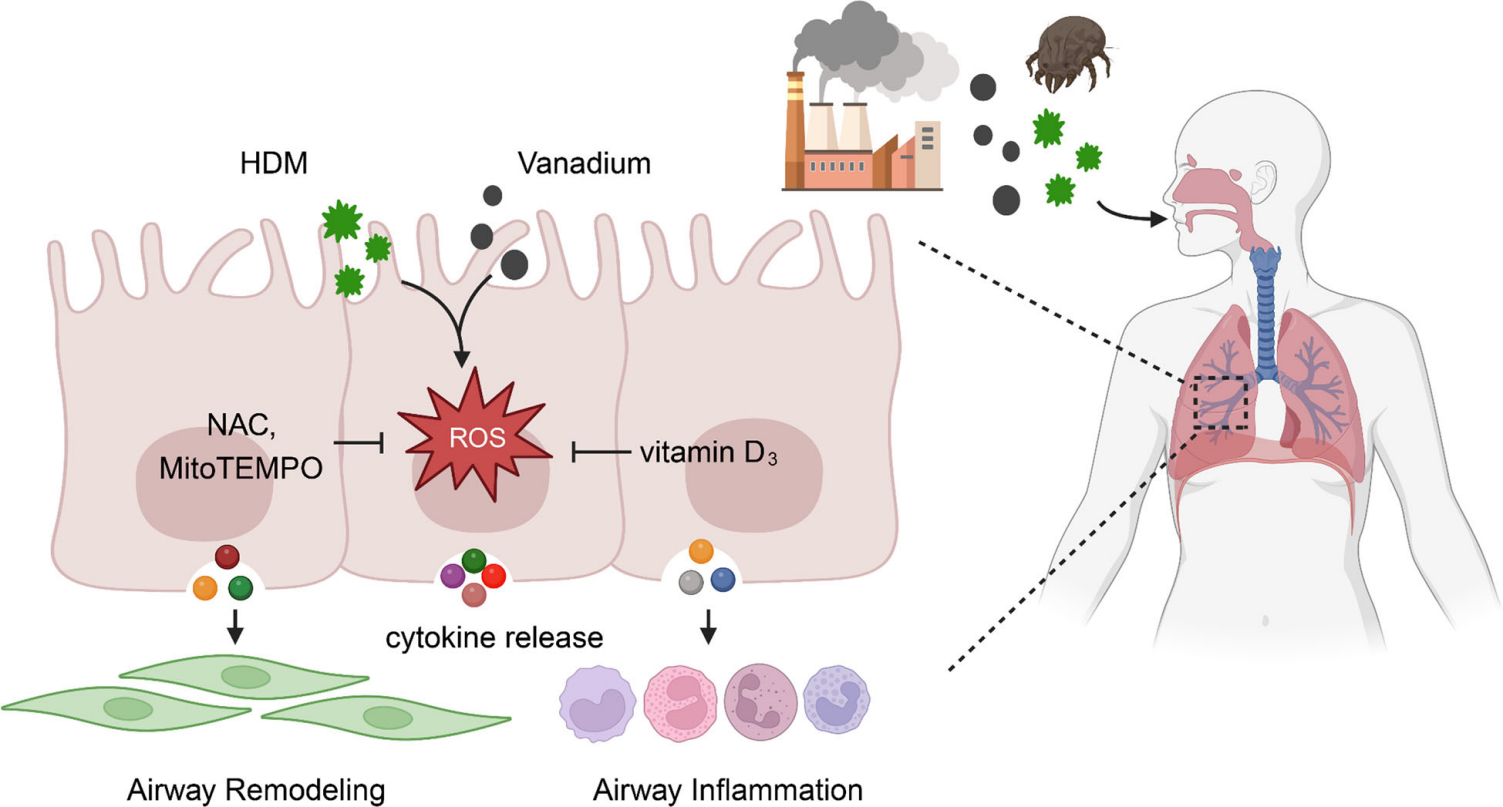
ROS functions as a molecular link for vanadium co-exposure with house dust mite (HDM) and epithelial cytokine release that contribute to airway inflammation and remodeling.

Taken together, we provide evidence that vanadium co-exposure can potentially potentiate and/or amplify HDM-induced allergic lung inflammation and airway remodeling. Mechanistically, we found that ROS function as a molecular link for vanadium co-exposure and epithelial cytokine release that contribute to the Th2-associated airway inflammation and airway remodeling (**Figure 8**). Of great relevance for the clinic, our finding reveals that ROS inhibitors and antioxidant VD_3_ can be used to prevent or treat environmental pollutant co-exposure promoted allergen-induced airway inflammation and remodeling. Our observations provide the basis for future study to investigate how ROS regulate airway inflammation and remodeling, and to further determine whether targeting ROS is an efficient strategy to prevent or treat environmental pollutant-mediated exacerbation of allergic airway inflammation and remodeling.

## Data availability statement

The raw data supporting the conclusions of this article will be made available by the authors, without undue reservation.

## Ethics statement

The experimental protocols were reviewed and approved by the animal Care and Use Committee in Shenzhen University and were in accordance with the guidelines and regulations of the institution.

## Author contributions

WT, XX, JL, XL, RY, RW, YS, and MG performed experiments, analyzed data, and review the manuscript. WT and PG wrote the manuscript. S-KH and PG designed and supervised the study, and wrote the manuscript. EW, DX, and PY provided intellectual input and aided in the experimental design. All authors contributed to the article and approved the submitted version.
